# Myeloid thrombomodulin lectin-like domain inhibits osteoclastogenesis and inflammatory bone loss

**DOI:** 10.1038/srep28340

**Published:** 2016-06-17

**Authors:** Tsung-Lin Cheng, Chao-Han Lai, Shyh-Jou Shieh, Yin-Bo Jou, Jwu-Lai Yeh, Ai-Lun Yang, Yan-Hsiung Wang, Chau-Zen Wang, Chung-Hwan Chen, Guey-Yueh Shi, Mei-Ling Ho, Hua-Lin Wu

**Affiliations:** 1 Department of Physiology, College of Medicine, Kaohsiung Medical University, Kaohsiung, Taiwan; 2 Orthopaedic Research Center, College of Medicine, Kaohsiung Medical University Hospital, Kaohsiung Medical University, Kaohsiung, Taiwan; 3 Cardiovascular Research Center, National Cheng Kung University, Tainan, Taiwan; 4 Department of Surgery, National Cheng Kung University Hospital, College of Medicine, National Cheng Kung University, Tainan, Taiwan; 5 International Research Center for Wound Repair and Regeneration (iWRR), National Cheng Kung University, Tainan, Taiwan; 6 Department of Biochemistry and Molecular Biology, College of Medicine, National Cheng Kung University, Tainan, Taiwan; 7 Department of Pharmacology, College of Medicine, Kaohsiung Medical University, Kaohsiung, Taiwan; 8 Graduate Institute of Medicine, College of Medicine, Kaohsiung Medical University, Taiwan; 9 Graduate Institute of Exercise Science, University of Taipei, Taipei, Taiwan; 10 School of Dentistry, College of Dental Medicine, Kaohsiung Medical University, Kaohsiung, Taiwan; 11 Department of Orthopedics, College of Medicine, Kaohsiung Medical University, Kaohsiung, Taiwan; 12 Department of Orthopedics, Kaohsiung Medical University Hospital, Kaohsiung Medical University, Kaohsiung, Taiwan; 13 Department of Orthopedics, Kaohsiung Municipal Ta-Tung Hospital, Kaohsiung Medical University, Kaohsiung, Taiwan; 14 Department of Marine Biotechnology and Resources, National Sun Yat-sen University, Taiwan

## Abstract

Osteoclastogenesis is an essential process during bone metabolism which can also be promoted by inflammatory signals. Thrombomodulin (TM), a transmembrane glycoprotein, exerts anti-inflammatory activities such as neutralization of proinflammatory high-mobility group box 1 (HMGB1) through TM lectin-like domain. This study aimed to identify the role of myeloid TM (i.e., endogenous TM expression on the myeloid lineage) in osteoclastogenesis and inflammatory bone loss. Using human peripheral blood mononuclear cells and mouse bone marrow-derived macrophages, we observed that the protein levels of TM were dramatically reduced as these cells differentiated into osteoclasts. In addition, osteoclastogenesis and extracellular HMGB1 accumulation were enhanced in primary cultured monocytes from myeloid-specific TM-deficient mice (LysMcre/TM^*flox/flox*^) and from TM lectin-like domain deleted mice (TM^*LeD/LeD*^) compared with their respective controls. Micro-computerized tomography scans showed that ovariectomy-induced bone loss was more pronounced in TM^*LeD/LeD*^ mice compared with controls. Finally, the inhibiting effects of recombinant TM lectin-like domain (rTMD1) on bone resorption *in vitro*, and bone loss in both the ovariectomized model and collagen antibody-induced arthritis model has been detected. These findings suggested that the myeloid TM lectin-like domain may inhibit osteoclastogenesis by reducing HMGB1 signaling, and rTMD1 may hold therapeutic potential for inflammatory bone loss.

Osteoclastogenesis is a critical process for bone resorption during bone metabolism. Precursor monocytes/macrophages can differentiate into multinuclear osteoclasts during osteoclastogenesis. Promotion of osteoclastogenesis requires two essential hematopoietic factors, i.e. macrophage colony stimulating factor (M-CSF) and receptor activator of nuclear factor kappa-B ligand (RANKL)^1^,^2^. Following activation of receptor activator of nuclear factor kappa-B (RANK) on the surface of precursor monocytes/macrophages, expression of several genes (e.g., tartrate-resistant acid phosphatase (TRAP), cathepsin K (CATK), calcitonin receptor, and β_3_integrin) can be induced in the osteoclasts^2^,^3^. Other cells such as activated T-cells can modulate the formation of osteoclasts by secreting RANKL or osteoprotegerin, suggesting that the immune system might affect bone metabolism and homeostasis. This interaction has led to an interdisciplinary research field known as osteoimmunology that investigates the crosstalk between immune/inflammatory responses and bone metabolism^4^.

Thrombomodulin (TM) was first discovered as a membrane glycoprotein that activates protein C as an anti-coagulant^5^. TM belongs to the C-type lectin-like domain superfamily, and is expressed in various cell types such as keratinocytes, endothelial cells, myeloid-derived monocytes and macrophages^6^,^7^,^8^. Previous studies have demonstrated that TM participates in various biological processes such as cell-cell adhesion, epithelial-mesenchymal transition, keratinocyte differentiation and cutaneous wound healing, and inflammation^9^,^10^,^11^,^12^. From the N- to C-terminus, TM is composed of five functional domains, including a C-type lectin-like domain (domain 1, TMD1), a domain with six epidermal growth factor (EGF)-like structures (domain 2, TMD2), a serine/threonine-rich domain (domain 3, TMD3), a transmembrane domain (domain 4, TMD4), and a cytoplasmic domain (domain 5, TMD5)^13^. The soluble forms of TM (sTM) have been considered a marker of organ dysfunction and a novel angiogenic factor^14^,^15^. The expression of TM is tightly regulated by inflammatory responses^16^. TM also exerts anti-inflammatory activities where TMD1 may sequester the pro-inflammatory high-mobility group box 1 (HMGB1) protein. This would prevent HMGB1 from engaging its receptors that may result in sustained chronic inflammatory responses and eventually in tissue damage^17^. In addition, TM aids the proteolytic cleavage of HMGB1 by thrombin^18^. Our previous study also demonstrated that recombinant TMD1 (rTMD1) suppresses inflammation by directly binding to lipopolysaccharide (LPS)^19^. Taken together, these studies reveal that TM functional domains can modulate inflammatory responses. Recently, endosialin (CD248), another member of the C-type lectin-like domain containing proteins, has been reported as a negative regulator of bone formation in mice^20^,^21^. However, the significance of TM during osteoclastogenesis is not clear.

Macrophages are derived from monocytes that represent circulating cell members of the myeloid lineage. In this study, we examined the hypothesis that the myeloid TM lectin-like domain negatively regulates osteoclastogenesis. RAW264.7 cells, a mouse macrophage cell line, and isolated human peripheral blood mononuclear cells (PBMCs) were used to identify the expression pattern of TM during osteoclastogenesis *in vitro*. In addition, the functions of TM and its lectin-like domain during the formation of osteoclasts were investigated using primary cultured monocytes/macrophages from the TM transgenic mice LysMcre/TM^*flox/flox*^ (myeloid-specific TM-deficient mice) and TM^*LeD/LeD*^ (TM lectin-like domain deleted mice), along with their respective controls. Furthermore, the potential mechanism by which TM suppresses osteoclastogenesis was explored. Finally, the therapeutic effects of rTMD1 on bone resorption *in vitro* and bone loss *in vivo* were evaluated. The results would elucidate the significance of myeloid TM and its lectin-like domain in osteoporosis.

## Results

### TM protein expression in monocytes/macrophages was reduced during osteoclastogenesis

**Figure 1:**
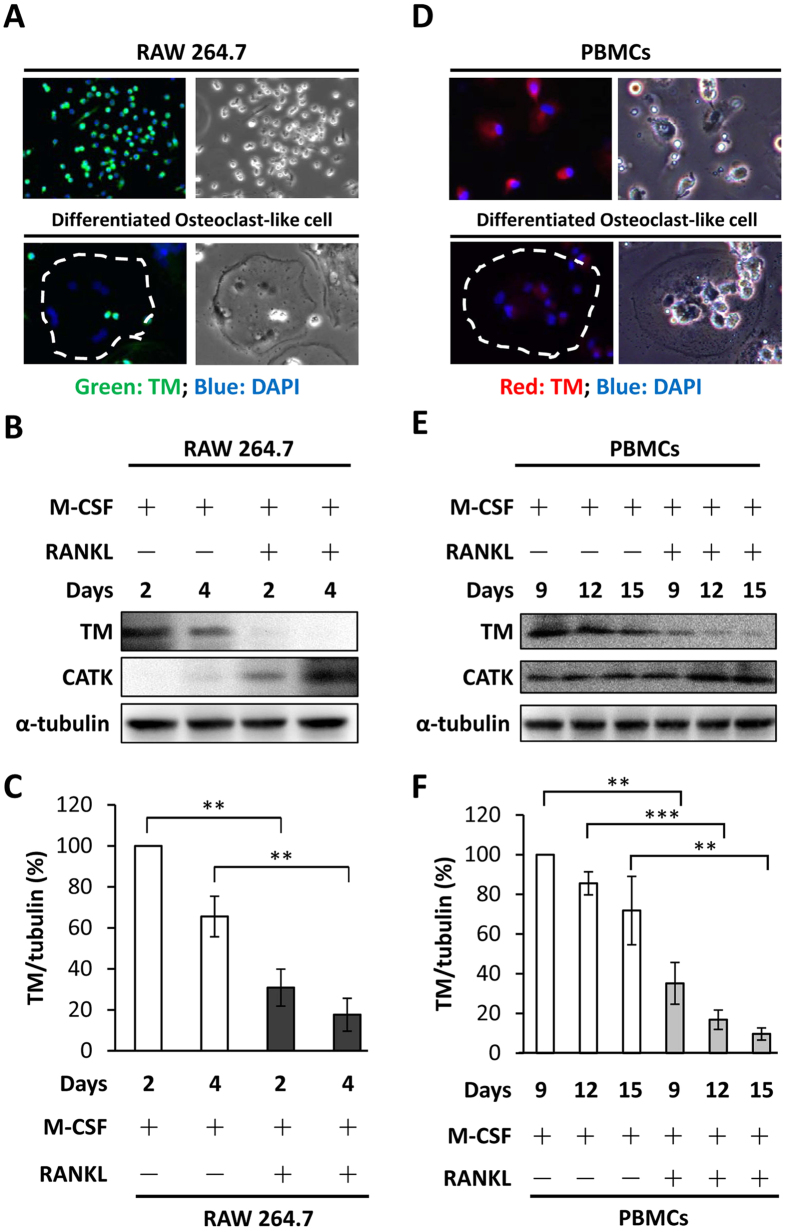
Down-regulated TM expression in mammalian osteoclast-like cells. Immunofluorescence staining of TM expression in RAW 264.7 cells, PBMCs and their differentiated osteoclast-like cells induced by treatment with M-CSF (20 ng/mL) and RANKL (30 ng/mL) for (**A**) 4 days and for (**D**) 1 week (original magnification × 200). Western blot analysis of TM and CATK expression in (**B**) RAW 264.7 cells and (**E**) PBMCs at indicated times after treatment with M-CSF and RANKL. Representative figures from three independent experiments are shown. (**C**,**F**) Quantitative representation of (**B**,**E**). Statistics were performed by Student’s t-test. **P < 0.01, ***P < 0.001.

Osteoclastogenesis in mouse RAW 264.7 cells and human PBMCs was induced using RANKL and M-CSF to evaluate the levels of TM protein during the process. Immunofluorescence staining revealed that TM expression was dramatically decreased as RAW 264.7 cells differentiated into osteoclast-like cells (Fig. 1A). Western blot analysis showed that treatment of RAW 264.7 cells with RANKL reduced TM but increased CATK, a marker of osteoclasts, in a time-dependent manner (Fig. 1B,C). Moreover, similar results were observed in human PBMCs (Fig. 1D–F). These results suggested that TM expression in monocytes/macrophages may be inversely related to osteoclastogenesis.

### Deficiency of full-length TM in macrophages enhanced the RANKL-induced osteoclastogenesis

**Figure 2:**
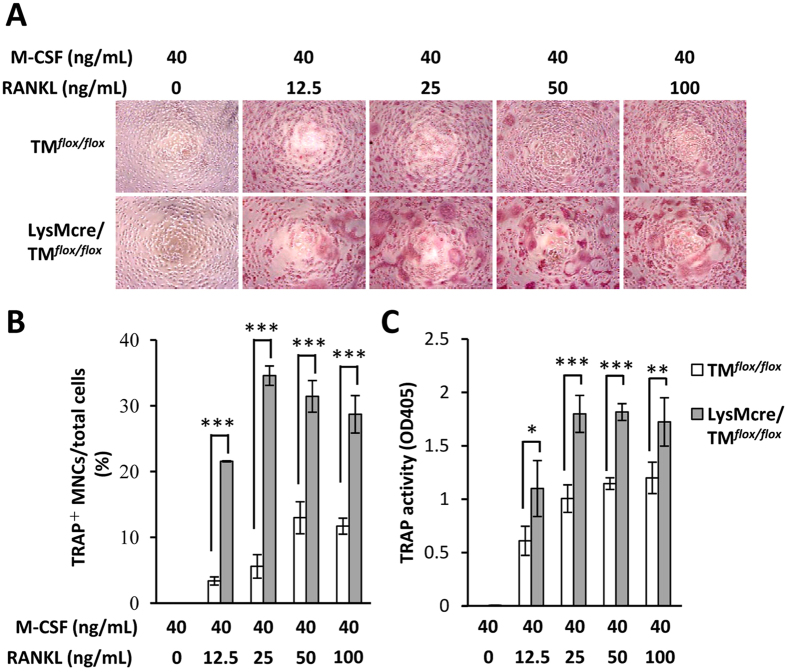
Myeloid-specific TM deletion enhanced osteoclasts formation. (**A**) TRAP staining of osteoclasts differentiated from primary cultured TM^*flox/flox*^ and LysMcre/TM^*flox/flox*^ mice macrophages, treated with M-CSF and various concentrations of RANKL for differentiation (original magnification × 200). (**B**) Calculated ratio of TRAP^+^ MNCs to total cells per well. (**C**) Quantitation of TRAP activity using 4-NPP as substrate. Statistics were performed by Two-way ANOVA. *P < 0.05, ***P < 0.001. Data showed the mean ± SD for three independent experiments.

To investigate whether TM might be a negative regulator in osteoclastogenesis, macrophages from myeloid-specific TM-deficient mice were isolated. TRAP staining showed that RANKL-induced osteoclastogenesis in LysMcre/TM^*flox/flox*^ macrophages was more effective than that from TM^*flox/flox*^ macrophages (Fig. 2A). Quantification of the results showed that the ratio of differentiated TRAP positive multinucleated cells (TRAP^+^ MNCs) in TM-deficient macrophages was at least 3-fold higher compared with those in TM-wildtype macrophages (Fig. 2B). Moreover, TRAP activities in TM-deficient macrophages were significantly higher than those in TM-wildtype macrophages (Fig. 2C). These results indicated that the expression of full-length TM in macrophages may hinder RANKL-induced osteoclastogenesis.

### RANKL-induced osteoclastogenesis inhibited by myeloid TM lectin-like domain

**Figure 3:**
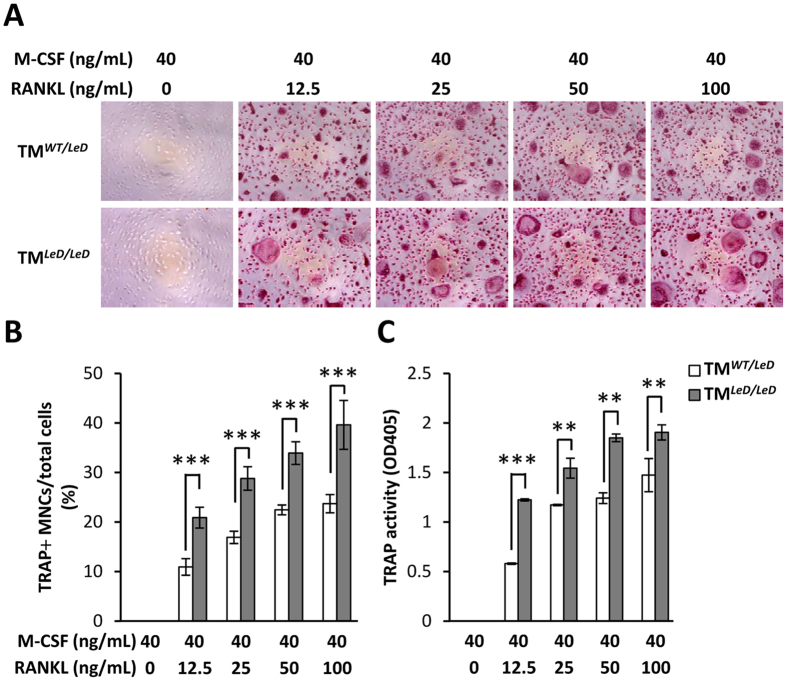
Inhibition of RANKL-induced osteoclast formation by TM lectin-like domain on macrophages. (**A**) TRAP staining of osteoclasts differentiated from primary cultured TM^*WT/LeD*^ and TM^*LeD/LeD*^ mice macrophages, treated with M-CSF and various concentrations of RANKL for differentiation (original magnification × 200). (**B**) Ratio of TRAP^+^ MNCs to total cells per well. (**C**) Quantitation of TRAP activity using 4-NPP as substrate. Statistics were performed by Two-way ANOVA. **P < 0.01, ***P < 0.001. Experiments were repeated three times.

It has been showed that osteoblast-derived C-type lectin could inhibit osteoclast formation^22^. To further investigative whether the lectin-like domain of myeloid TM contributed to RANKL-induced osteoclastogenesis inhibition, primary macrophages from TM lectin-like domain-deficient TM^*LeD/LeD*^ mice and their controls TM^*WT/LeD*^ mice were obtained. TRAP staining showed that RANKL-induced osteoclastogenesis was more prominent in macrophages from TM^*LeD/LeD*^ mice than those in TM^*WT/LeD*^ mice (Fig. 3A). Also, the TRAP^+^ MNCs ratio and TRAP activities in TM lectin-like domain-deficient macrophages were significantly higher than those in controls (Fig. 3B,C). These results suggested that the lectin-like domain of myeloid TM may have a critical effect on the inhibition of RANKL-induced osteoclastogenesis.

### Deletion of the full-length TM or TM lectin-like domain enhanced HMGB1 secretion and bone resorption in osteoclasts and increased OVX-induced serum HMGB1 level and bone loss

**Figure 4:**
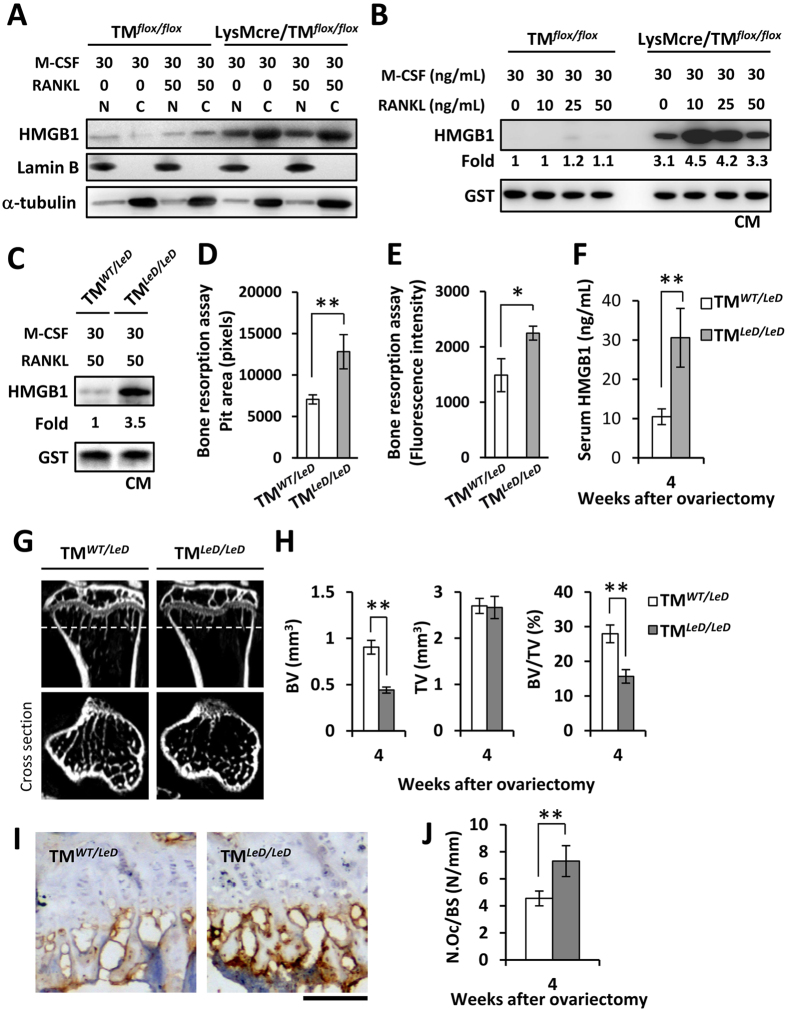
TM deficiency enhanced HMGB1 translocation and secretion, bone resorption, and ovariectomy-induced bone loss. (**A**) After 24-hour treatment, whole-cell lysates were fractionated into nuclear (**N**) and cytosolic (**C**) fractions. The localization of HMGB1 was analyzed by western blotting. Evaluation of HMGB1 secretion by western blot analysis in primary cultured macrophages from (**B**) TM^*flox/flox*^ and LysMcre/TM^*flox/flox*^ mice, and from (**C**) TM^*WT/LeD*^ and TM^*LeD/LeD*^ mice. GST was added as internal control. CM, conditioned medium. Results of bone resorption assay were obtained by measuring the (**D**) pit area in fluoresceinamine-labeled chondroitin sulfate/calcium phosphate-coated plates and (**E**) fluorescence intensity in conditioned medium. (**F**) Detected HMGB1 levels in serum by ELISA assay after 4 weeks of ovariectomy. (**G**) Bone loss in tibia was detected by μCT scanning after 4 weeks of ovariectomy. Dashed lines indicated the cross sections. (**H**) Quantitative results of trabecular bone volume (BV), total bone volume (TV), and BV/TV in (**G**). n = 5 each group. (**I**) Immunohistochemical staining for cathepsin K in the tibia (brown). bar = 100 μm. (**J**) Osteoclast number/bone surface (N.Oc/BS, N/mm) were measured. Statistics were performed by Student’s t-test. *P < 0.05, **P < 0.01.

Since HMGB1 release may be critical for osteoclastogenesis and rheumatic diseases^23^,^24^, we further evaluated the effects of TM deficiency on HMGB1 translocation, secretion, and bone loss. In TM^*flox/flox*^ cells, RANKL treatment enhanced cytoplasmic translocation of HMGB1 in the presence of M-CSF (Fig. 4A). The cytoplasmic fraction and total expression level of HMGB1 were increased in LysMcre/TM^*flox/flox*^ cells compared with TM^*flox/flox*^ cells while treated with M-CSF only. Similar results were observed when LysMcre/TM^*flox/flox*^ cells and TM^*flox/flox*^ were treated with M-CSF and RANKL, suggesting that full-length TM deletion may contribute to HMGB1 translocation and production in osteoclasts. In addition, RANKL-enhanced HMGB1 production in LysMcre/TM^*flox/flox*^ and TM^*LeD/LeD*^ cells increased more than 3-fold compared with their respective controls (Fig. 4B,C). Bone resorption activities, as indicated by pit area and fluorescence intensity, were significantly enhanced in isolated TM^*LeD/LeD*^ cells compared with TM^*WT/LeD*^ cells (Fig. 4D,E). Similarly, serum HMGB1 level in TM^*LeD/LeD*^ was significantly higher than that in TM^*WT/LeD*^ mice (Fig. 4F). In addition, μCT scanning showed that OVX-induced tibia bone loss was significantly more severe in TM^*LeD/LeD*^ mice than in TM^*WT/LeD*^ mice (Fig. 4G,H). The expression levels of CATK and the number of osteoclasts were elevated in the bone section of TM^*LeD/LeD*^ mice as compared with TM^*WT/LeD*^ mice (Fig. 4I,J). These data suggested that deficiency of myeloid TM lectin-like domain led to enhanced bone resorption and bone loss, probably in association with extracellular HMGB1 production.

### Treatment with rTMD1 inhibited bone resorption and OVX-induced bone loss

**Figure 5:**
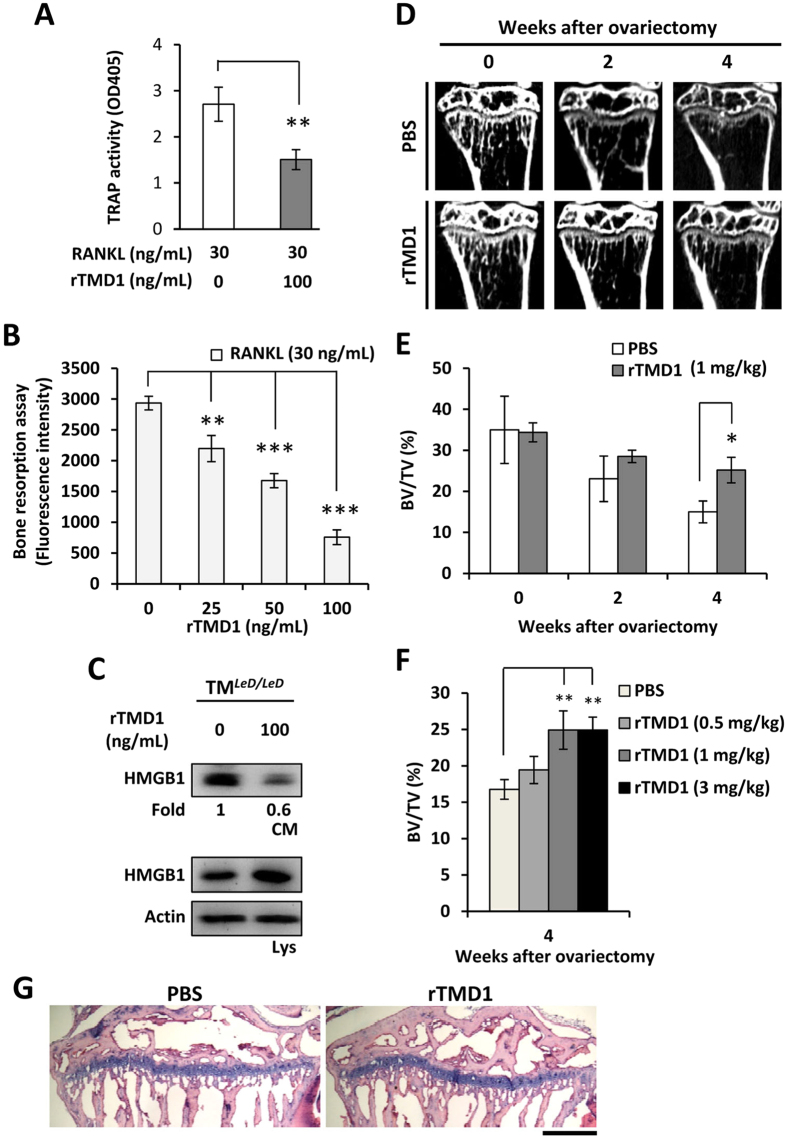
Recombinant TM lectin-like domain (rTMD1) suppressed RANKL-induced bone resorption and reduced ovariectomy-induced bone loss. (**A**) RANKL-induced TRAP activity in RAW264.7 cells was measured after rTMD1 treatment for 4 days. (**B**) Results of RANKL-induced bone resorption in RAW264.7 cells after treatment with various doses of rTMD1. (**C**) Western blotting analysis of extracellular HMGB1 from TM^*LeD/LeD*^ mice BMMs in response to rTMD1 treatment. CM, conditioned medium; Lys, cell lysates. (**D**) After OVX, an intraperitoneal injection of rTMD1 (1 mg/kg) was performed every 3 days until sacrificed. Bone loss in tibia was detected by μCT scanning in wild-type mice with or without rTMD1 treatment, within 4 weeks of ovariectomy. (**E**) Quantified BV/TV of (**D**). n = 5. (**F**) Quantitative results of BV/TV in ovariectomized wild-type mice treated with various doses of intraperitoneal rTMD1 injection (0.5 mg/kg, 1 mg/kg, 3 mg/kg). n = 5. BV, trabecular bone volume; TV, total bone volume. (**G**) H&E staining of the tibia after 4 weeks of ovariectomy. bar = 50 μm. Student’s t-test (**A**); One-way ANOVA (**B**), (**F**); Two-way ANOVA (E). *P < 0.05, **P < 0.01, ***P < 0.001.

TMD1 has been demonstrated to neutralize HMGB1-triggered inflammation^17^. Therefore, we investigated the effects of human rTMD1 on bone resorption *in vitro* and bone loss *in vivo*. Treatment with rTMD1 in RAW264.7 cells significantly inhibited RANKL-induced TRAP activity (Fig. 5A) and dose-dependently reduced bone resorption activity (Fig. 5B). Furthermore, rTMD1 treatment also attenuated extracellular HMGB1 production in TM^*LeD/LeD*^ cells (Fig. 5C). *In vivo*, rTMD1 treatment suppressed bone loss across a 4-week period and increased the bone volume fraction (BV/TV, %) in a dose-dependent manner (Fig. 5D–F). Furthermore, histomorphometric analysis showed that the bone loss was reduced by treatment with rTMD1 (Fig. 5G). These results suggested that rTMD1 inhibited bone loss, at least in part, through blockade of HMGB1-induced osteoclastogenesis.

### Treatment with rTMD1 inhibited bone loss in CAIA mice

**Figure 6:**
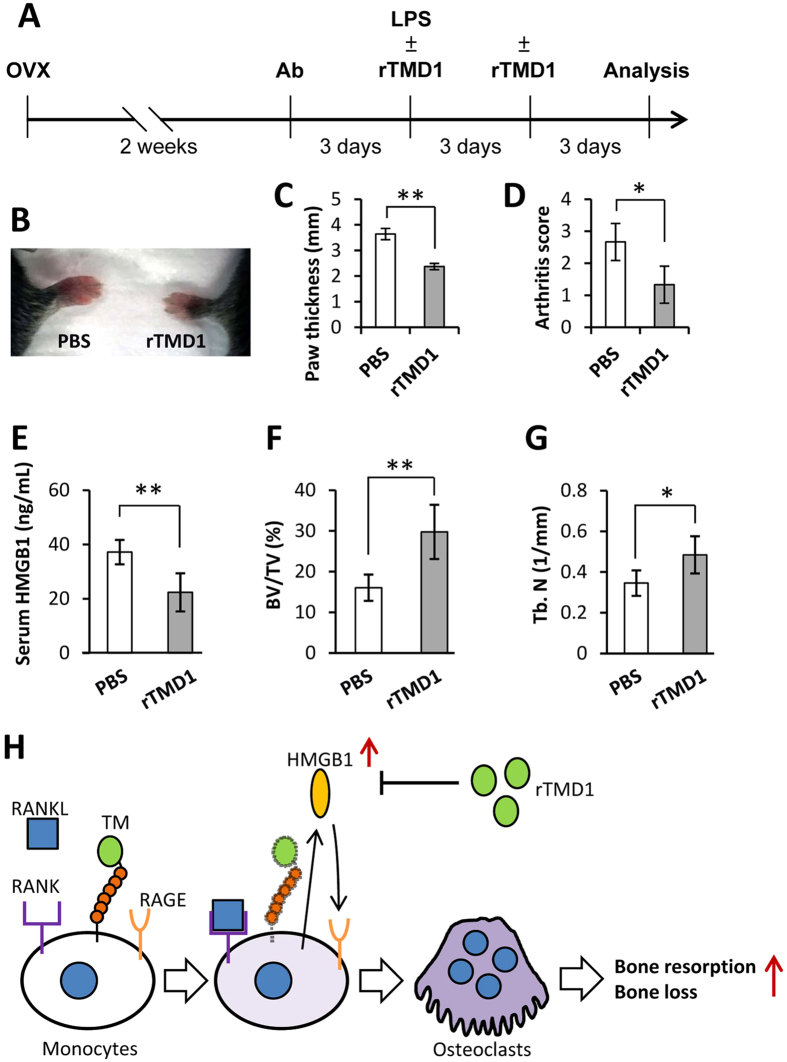
Treatment with rTMD1 inhibited bone loss and reduced serum HMGB1 in CAIA mouse model. (**A**) Experimental design of CAIA and rTMD1 treatment (1 mg/kg). (**B**) The front paws of CAIA mice with and without rTMD1 treatment. (**C**) Paw thickness. (**D**) Arthritis score. (**E**) Results of HMGB1 serum levels in mouse with or without rTMD1 treatment. (**F**) Quantitative results of BV/TV. (**G**) Quantitative results of Tb. N. n = 3. Student’s t-test (**E**–**G**). *P < 0.05, **P < 0.01. (**H**) A schematic model of myeloid TM in osteoclastogenesis and inflammatory bone loss. During osteoclastogenesis, RANKL reduced TM expression and promoted extracellular HMGB1 secretion in monocytes, which resulted in the enhancement of bone resorption and bone loss. In addition, rTMD1 treatment could inhibit HMGB1 secretion, bone resorption, and bone loss.

Finally, the therapeutic effects of rTMD1 treatment in the CAIA model, a model featured with joint inflammation and generalized bone loss^25^, were also evaluated. The experimental design was showed in Fig. 6A. Our results showed that rTMD1 treatment significantly reduced the paw thickness (Fig. 6B,C), arthritis score (Fig. 6D), and levels of serum HMGB1 (Fig. 6E). Compared with mice treated with PBS, those supplemented with rTMD1 had significantly higher bone volume fraction (BV/TV, %) (Fig. 6F) and trabecular bone number (Tb. N, 1/mm) (Fig. 6G). Taken together, these data suggested that rTMD1 treatment may inhibit inflammatory bone loss in the CAIA model.

## Discussion

Osteoporosis is a disorder caused by increased bone resorption and reduced bone formation^26^. Normal bone resorption coupled with reduced synthesis of bone matrix may be considered as low-turnover osteoporosis. In contrast, high-turnover osteoporosis indicates a condition where increased bone resorption results from increased osteoclast activity. Low-turnover osteoporosis is generally induced by corticosteroids, whereas high-turnover osteoporosis is associated with estrogen deficiency or rheumatoid arthritis and other chronic inflammatory disorders^27^,^28^.

In this study, we demonstrated that myeloid TM was reduced during osteoclastogenesis (Fig. 1), implicating a role of myeloid TM in high-turnover osteoporosis. RANKL-induced osteoclastogenesis was promoted in full-length TM-deficient macrophages. In addition, RANKL-induced osteoclastogenesis was promoted in macrophages with specific deletion of TM lectin-like domain. These observations suggested that full-length TM in macrophages may inhibit osteoclastogenesis, and that this inhibitory effect likely resulted from its lectin-like domain.

RANKL is one of the key regulatory molecules in osteoclast formation through activation of intracellular NF-κB signaling in osteoclast precursor cells. NF-κB is a critical mediator of TM repression by cytokines^29^. Therefore, it is plausible that through the activation of NF-κB, RANKL induced TM down-regulation in monocytes/macrophages. In our study, genetic deletion of TM in monocytes/macrophages led to the promotion of osteoclastogenesis (Figs 2 and 3). Previous reports have demonstrated that long-standing diabetes results in osteoporosis in rats, and diabetic patients have a high risk for osteoporotic fractures^30^,^31^. Other studies have indicated that a high-glucose environment may activate NF-κB, which may suppress TM expression^32^,^33^. These findings suggested that diseases with suppressed TM expression may increase osteoporosis risk. Along with these findings, our study may provide a new perspective in explaining diabetes-related bone loss.

Extracellular HMGB1, which can be actively released by activated monocytes/macrophages, is a critical factor for RANKL-induced osteoclastogenesis^23^,^34^. HMGB1 is also involved in a positive feedback mechanism to sustain inflammation^35^. In the present study, myeloid-specific TM knockout enhanced extracellular HMGB1 production *in vitro*. Similarly, increased HMGB1 secretion, bone resorption, and bone loss were also observed in mice with whole-body TM lectin-like domain deletion (Fig. 4). These suggesting that the mechanisms by which TM regulates osteoclastogenesis and bone remodeling may involve, at least in part, the inhibition of extracellular HMGB1 production. Moreover, rTMD1 treatment reduced the extracellular HMGB1 levels and bone loss not only in the ovariectomized model, but also in the CAIA model (Figs 5 and 6). This is consistent with a previous study that showed rTMD1 suppressed inflammation and arthritis in mouse model^36^.

HMGB1 deficient and conditional knockout mice have been generated in previous studies. In one study, HMGB1-deficient mice may die within 24 hours after birth due to insufficient glucocorticoid receptor expression and hypoglycaemia^37^. In the other study, results obtained from HMGB1 conditional knockout mice showed that myeloid HMGB1 contributes to protection from endotoxemia and bacterial infection in mice^38^. Based on the observations in our study, further studies using HMGB1 conditional knockout mice to study the definitive role of HMGB1 in osteoclastogenesis and bone remodeling are warranted.

Estrogen replacement is the traditional therapy used to prevent bone loss and fractures^39^. However, the risk of breast cancer may be accentuated following estrogen therapy^40^. Currently, other therapeutic approaches are developed to inhibit excessive bone resorption, such as bisphosphonates or antibodies of RANKL or CATK, while intermittent administration of parathyroid hormone is developed to promote bone formation^41^,^42^. In our study, rTMD1 suppresses osteoclastogenesis and holds promise as an effective therapeutic agent for osteoporosis.

In summary, we demonstrated for the first time that myeloid TM lectin-like domain functions as a key inhibitor of HMGB1-mediated osteoclastogenesis (Fig. 6E). Therefore, administration of recombinant TM lectin-like domain may hold therapeutic potential for postmenopausal osteoporosis or inflammation-related bone loss.

## Methods

### Reagents

Antibodies and recombinant proteins were purchased from the following companies: anti-tubulin antibody (Calbiochem EMD Biosciences, La Jolla, California, USA); anti-TRAP antibody (Invitrogen Carlsbad, CA, USA); 4-nitrophenyl phosphate (4-NPP), anti-TM antibody, anti-cathepsin K (CATK) antibody, anti-actin antibody, anti-glutathione S-transferase (GST) antibody, anti-lamin B antibody, and RANKL protein (Santa Cruz, CA, USA); ArthritoMab™ CII mAb cocktail (MD Biosciences GmbH, Zürich, Switzerland); and M-CSF protein (R&D System, Minneapolis, MN, USA). Lipopolysaccharide (LPS) was purchased from MD Biosciences. RAW264.7 cells were purchased from the American Type Culture Collection (ATCC, Rockville, MD, USA). The TM domains were prepared in our laboratory as previously described^43^.

### Experimental animals

LysMcre/TM^*flox/flox*^ mice with myeloid-specific TM deletion were produced as previously reported^9^. LysMcre/TM^*flox/flox*^ mice had their TM expression deletion in the myeloid lineage except for other tissues and organs, whereas TM^*flox/flox*^ mice had no TM suppression. Mice lacking the lectin-like domain of TM (TM^*LeD/LeD*^ mice) and their controls (TM^*WT/LeD*^ mice) were a gift from Dr. Conway^44^. These B6-background mice were maintained in a pathogen-free animal facility at the Animal Center of National Cheng Kung University (NCKU). Collagen antibody-induced arthritis (CAIA) was performed in C57BL/6 mice, along with bilateral ovariectomy (OVX) and evaluation of the paw thickness and arthritis scores as previously reported^25^. OVX was performed at 8 weeks of age. Histomorphometric analysis of the tibia was performed on paraffin sections after decalcification, and the number of osteoclasts/bone surface (N.Oc/BS, N/mm) was counted as previously described^45^. All animal protocols were approved by the Institutional Animal Care and Use Committee of NCKU. The methods were carried out in accordance with the approved guidelines.

### Cell isolation and cell culture

Human PBMCs were separated from whole blood using a Ficoll-Paque PLUS (GE Healthcare, Brussels, Belgium) gradient based on the manufacturer’s instructions. Appropriate informed consent was obtained from the volunteers. The study protocols and consent documents were approved by the Institutional Review Board of NCKU Hospital. Mouse bone marrow macrophages/monocytes (BMMs) were isolated from the femur and tibia (6 to 12 weeks old) as previously described with some modifications^46^. Briefly, isolated total bone marrow cells were incubated overnight in complete Minimum Essential Medium (Alpha modification; α-MEM). Subsequently, nonadherent cells were collected and mononuclear cells were prepared using Ficoll-Hypaque (GE Healthcare, Piscataway, NJ, USA) density gradient centrifugation. The BMMs were evenly scattered across the interface between Ficoll-Hypaque and medium. Cells were cultured in α-MEM supplemented with 5% heat-inactivated fetal bovine serum (FBS), L-glutamine (292  mg/L), and 1% antibiotics (penicillin and streptomycin; Invitrogen, Carlsbad, CA, USA). The methods were carried out in accordance with the approved guidelines.

### Osteoclast culture, conditioned medium collection, TRAP staining

Cells (RAW264.7, PBMCs, or BMMs) were cultured in 96-well plates (1  ×  10^5^ cells/well). RANKL and M-CSF were added to stimulate osteoclast generation. Media were replenished every 2 days. After 24 hours of treatment, the conditioned medium was concentrated using Centricon tubes (Amicon, Beverly, MA), and GST was added (20 μg per sample) as an internal control. The concentrated samples were then subjected to western blot analysis. On day 4 or day 7, cells were fixed with 3% formaldehyde and were stained with TRAP (Sigma, St. Louis, MO). The ratio of TRAP-positive multinucleated cells (>4 nuclei/cell) to total cells in each well was calculated^47^.

### Subcellular fractionation

Subcellular fractionation was performed following the protocol by Abcam (http://www.abcam.com/ps/pdf/protocols/subcellular_fractionation.pdf). Briefly, cells were lysed with a subcellular fractionation buffer and cell lysates were centrifuged. After centrifuge, the pellet was resuspended in nuclear buffer and the nuclear fraction was obtained. The supernatant was re-centrifuged at a higher speed (40,000 rpm). The cytoplasmic fraction was obtained after ultra-centrifugation.

### TRAP activity assay

TRAP activity was measured using 4-NPP as substrate, based on the microplate assay method with modifications^48^. A 50 μL cell lysate was incubated with 150 μL of substrate (8 mM 4-NPP, 100 mM sodium acetate and 500 mM sodium tartrate, pH 5.0) for 40 min at 37 °C. The reaction was terminated by the addition of 50 μL 3 M NaOH. Absorbance was measured at 405 nm in a microplate reader (SPECTRAmax™340; Molecular Devices, Palo Alto, CA).

### Western blot analysis

Cells were lysed and western blot analysis was performed as previously described^49^. Approximately 50 μg of total protein was separated on 10% sodium dodecyl sulfate-polyacrylamide gel and transferred onto a polyvinylidene difluoride membrane (Amersham Pharmacia Biotech, Buckinghamshire, UK). After probing with a primary and a secondary antibody, the signal was detected using an enhanced chemiluminescence reagent (Amersham Pharmacia Biotech), and results were quantified using ImageJ software (National Institutes of Health, Bethesda, MD).

### Bone resorption assay

Bone resorption activities were determined by the bone resorption assay kit (Cosmo Bio. Co. Ltd., Tokyo, Japan) based on the manufacturer’s instructions. In brief, cells were incubated on fluoresceinated calcium phosphate-coated microplate with M-CSF (30 ng/mL) and RANKL (30 ng/mL) in the presence or absence of various concentrations of rTMD1 for 7 days. RANKL-induced bone resorption activity was evaluated by measuring pit area of each well, and by detecting the fluorescence intensity of conditioned medium at an excitation wavelength of 485 nm and an emission wavelength of 535 nm.

### Quantification of serum HMGB1

Serum levels of HMGB1 were detected from 10 μL sera using HMGB1 ELISA kit II (Shino-Test Corp., Kanagawa, Japan) according to the manufacturer’s instructions.

### Micro-Computed Tomography (μCT)

All μCT analyses performed in this study were consistent with the current guidelines^50^. Bone samples from all groups were imaged using a SkyScan-1076 Micro-CT System (Skyscan, Kontich, Belgium). For trabecular bone analysis, the μCT scanner was operated at 45 kV, 220 μA, 0.4 μ rotation step, 0.5 mm aluminum filter, and a scan resolution of 18 μm/pixel. The following 3D parameters were measured by software CT Analyser (Belgium): total bone volume (TV, mm^3^), trabecular bone volume (BV, mm^3^), trabecular bone volume fraction (BV/TV, %), and trabecular bone number (Tb. N, 1/mm).

### Statistical analysis

Data were expressed as mean ± standard deviation (SD). Student’s t test or analysis of variance (ANOVA) was used to assess the statistical significance for the respective data. Asterisks in the figures were used to indicate the levels of significance: *p  < 0.05, **p  < 0.005, and ***p  < 0.001.

## Additional Information

**How to cite this article**: Cheng, T.-L. *et al*. Myeloid thrombomodulin lectin-like domain inhibits osteoclastogenesis and inflammatory bone loss. *Sci. Rep.* **6**, 28340; doi: 10.1038/srep28340 (2016).
